# Cytotoxic and Pro-apoptotic Effects of Curcumin and Vitamin D₃ in a Human Non-Hodgkin’s Lymphoma Cell Line (Daudi): An In Vitro Study

**DOI:** 10.7759/cureus.95966

**Published:** 2025-11-02

**Authors:** Annuja Anandaradje, Rahini Rajendran, Anitha S, Alladi Charanraj Goud, Prasanth Ganesan, Jayanthi Mathaiyan, Sandhiya Selvarajan

**Affiliations:** 1 Clinical Pharmacology, Jawaharlal Institute of Postgraduate Medical Education and Research, Puducherry, IND; 2 Pharmacology, Jawaharlal Institute of Postgraduate Medical Education and Research, Puducherry, IND; 3 Institute of Molecular and Translational Medicine, Faculty of Medicine and Dentistry at Palacký University/University Hospital Olomouc, Olomouc, CZE; 4 Medical Oncology, Jawaharlal Institute of Postgraduate Medical Education and Research, Puducherry, IND

**Keywords:** apoptosis, combination therapy, curcumin, daudi cell, non-hodgkin’s lymphoma, vitamin d₃

## Abstract

Background: Non-Hodgkin’s lymphoma (NHL) is a heterogeneous group of lymphoid malignancies, with B-cell lymphomas being the most common. Despite advances in conventional therapies, adverse effects and resistance remain major limitations, prompting interest in safer alternatives. Curcumin, a polyphenolic compound from *Curcuma longa*, and vitamin D₃ (1,25-dihydroxyvitamin D₃) are natural agents with documented anticancer and pro-apoptotic effects. However, their combined effects in B-cell NHL are underexplored.

Methods: This in vitro study evaluated the cytotoxic and pro-apoptotic effects of curcumin and vitamin D₃, individually and in combination, on Daudi cells (a human Burkitt’s lymphoma cell line). Cytotoxicity was assessed by MTT assay. Apoptosis was confirmed by DAPI nuclear staining. Quantitative reverse transcription polymerase chain reaction (RT-PCR) was performed to analyse the expression of apoptosis and cell cycle regulatory genes (*Bcl-2*, *Bak*, *p21*, *p53*, *caspase-3*, *caspase-8*, *caspase-9*), normalised to GAPDH.

Results: Curcumin (IC₅₀ = 1.6 µM ± 0.16) and vitamin D₃ (IC₅₀ =15 µM ± 1.12) exhibited dose-dependent cytotoxicity. Combination treatment at lower doses (curcumin 0.5 µM + vitamin D₃ 7.5 µM) produced a synergistic effect (combination index 0.81). DAPI staining confirmed apoptosis in treated cells. Gene expression analysis revealed marked downregulation of the anti-apoptotic gene *Bcl-2*, and significant upregulation of pro-apoptotic genes *Bak*, *caspase-3*, *caspase-8*, and *caspase-9*. Combination therapy notably enhanced *Bak* (6.4-fold), *p53* (5.6-fold), and *p21* (2.2-fold) expression compared to monotherapies, suggesting activation of both intrinsic and extrinsic apoptotic pathways.

Conclusion: Curcumin and vitamin D₃ demonstrated cytotoxic, pro-apoptotic effects in Daudi cells, both independently and in combination. Preclinical and clinical studies are warranted to validate these effects and establish translational applications.

## Introduction

Non-Hodgkin's lymphoma (NHL), arising from B cells, T cells, or natural killer (NK) cells, is one of the most common haematological malignancies globally [1]. Though treatments including chemotherapy, radiation, monoclonal antibodies, stem cell transplants, etc., have enhanced overall survival, the resultant adverse effects, drug resistance, and higher recurrences underscore the need for novel, efficacious, as well as safe compounds [2]. Recently, there has been an overwhelming interest in exploring the potential of natural products for anticancer activity.

Plant products seem to affect targeting several signalling pathways implicated in cancer development with relatively diminutive toxicity toward normal cells [3]. Curcumin, a naturally occurring polyphenolic compound derived from the rhizome of *Curcuma longa* (turmeric), with its potent anticancer, anti-inflammatory, antioxidant, and immunomodulatory properties, has drawn a lot of attention [4]. In addition, a plethora of preclinical studies in cancer, including haematological malignancies, have demonstrated the effect of curcumin in suppressing angiogenesis, metastasis, and apoptosis [5]. It has shown control over various molecular targets, such as NF-κB (nuclear factor-kappa B), p53 (tumour protein p53), Bcl-2 (B-cell lymphoma 2) family proteins, caspases, and cyclin-dependent kinases in modulating cell cycle arrest and activating intrinsic and extrinsic apoptotic cascades [6,7].

Current research indicates a role for vitamin D_3_ in controlling cell proliferation, differentiation, and apoptosis in different malignancies, including lymphoid neoplasms [8]. Vitamin D_3,_ also known as cholecalciferol, binds to the vitamin D receptor (VDR), a nuclear transcription factor, and produces downstream effects like cell cycle arrest at the G1 phase, induction of pro-apoptotic genes (e.g., *Bax, p21, p53*), and suppression of anti-apoptotic genes (e.g., *Bcl2*) [9,10]. In addition, epidemiological evidences show an association of vitamin D deficiency with enhanced cancer risk, including lymphomas [11].

Considering the pro-apoptotic potential of curcumin and vitamin D_3_ in cancer cells, studying their individual and combined effects could be of benefit in the treatment of various malignancies [12]. Hence, the study aimed to evaluate the individual as well as combined effects of curcumin and vitamin D_3_ on cytotoxicity and pro-apoptosis in Daudi cell lines. 

## Materials and methods

Cell culture and treatment

The study was conducted at the Department of Clinical Pharmacology, Jawaharlal Institute of Postgraduate Medical Education and Research, Puducherry, India, from May 2023 to March 2024. Daudi cells (a human Burkitt’s lymphoma cell line) were obtained from the National Centre for Cell Science, Pune, Maharashtra, India (accession number: CCL-213).

Cells were cultured in RPMI-1640 medium (Gibco™; Thermo Fisher Scientific Inc., Waltham, Massachusetts, United States) supplemented with 10% foetal bovine serum (FBS) (Gibco™), 1% penicillin-streptomycin (CAS No.: 8025-06-7, Gibco™), and maintained at 37°C in a humidified incubator with 5% CO₂. For treatment, cells were seeded at a density of 1 × 10⁶ cells/mL and incubated with various concentrations of curcumin (CAS No.: 458-37-7; Merck/MilliporeSigma, Burlington, Massachusetts, United States) and/or vitamin D_3_ (1,25-dihydroxyvitamin D) (CAS No.: 67-97-0; Merck/MilliporeSigma) for 24 hours. Control cells were treated with an equivalent volume of dimethyl sulphoxide (DMSO) (CAS No.: 67-68-5; Merck/MilliporeSigma) (<0.1%).

Cytotoxicity assay

Cytotoxicity was assessed in triplicate using the MTT (3-(4,5-dimethylthiazol-2-yl)-2,5-diphenyltetrazolium bromide) assay. Briefly, Daudi cells were seeded in 96-well plates at a density of 3 × 10^5^ cells/well and treated with different concentrations of curcumin and vitamin D_3_, alone or in combination, for 24 hours. After treatment, 10 µL of MTT solution (5 mg/mL in phosphate-buffered saline (PBS)) was added to each well and incubated for four hours at 37°C. The formazan crystals formed were dissolved by adding 100 µL of DMSO, and absorbance was measured at 570 nm using a microplate reader (Awareness Technology Inc., Palm City, Florida, United Kingdom). Cytotoxicity was expressed as a percentage (%) relative to untreated control cells.

Nuclear staining

DAPI (4′,6-diamidino-2-phenylindole) nuclear staining [13] was performed to evaluate morphological changes in the nuclei associated with apoptosis. Treated and untreated Daudi cells were collected and washed twice with PBS. Of the dye mixture, 10 μL (100 μg/L) was added to 90 μL of the cell suspension. A drop of the stained cell suspension was placed on a glass slide, covered with a coverslip, and immediately observed under a fluorescence microscope (Axiocam; Zeiss, Oberkochen, Baden-Württemberg, Germany) at λ=405nm. Apoptotic cells showed condensed and fragmented nuclei, which appeared irregularly stained with DAPI.

RNA Isolation and quantitative polymerase chain reaction (q-PCR)

Total RNA was extracted from treated and control cells using Trizol reagent (CAS No.: 593-84-0; Invitrogen, Waltham, Massachusetts, United States) following the manufacturer’s protocol. RNA concentration and purity were assessed using a bio-spectrophotometer (Eppendorf SE, Hamburg, Germany). One microgram of RNA was reverse-transcribed into cDNA using a high-capacity cDNA reverse transcription kit (Thermo Fisher Scientific Inc.). q-PCR was performed using SYBR Green Master Mix (Thermo Fisher Scientific Inc.) on a real-time PCR system (Applied Biosystems, Waltham, Massachusetts, United States). Gene-specific primers were used for the amplification of p53, p21, *Bcl-2, Bak, caspase-3, caspase-8, caspase-9*, and housekeeping gene Glyceraldehyde-3-Phosphate Dehydrogenase (*GAPDH*). Primer sequences used in this study are listed in Table 1.

**Table 1:** List of primer sequences

Gene	Forward Primer (5'→3')	Reverse Primer (5'→3')
p53	CCTCAGCATCTTATCCGAGTGG	TGGATGGTGGTACAGTCAGAGC
p21	GTGGACCTGTCACTGTCTTGT	GGATTAGGGCTTCCTCTTGG
Bcl-2	GGTGAACTGGGGGAGGATTGT	GGAGAAATCAAACAGAGGCCG
Bak	CAGGACGACATGAACCGACC	GATGATGCAGGCGATTTGGT
Caspase-3	CATGGAAGCGAATCAATGGACT	CTGTACCAGACCGAGATGTCA
Caspase-8	GTTGGAAGGAGGAAGCAGTG	CAGACTTCTGCTGCCATCTT
Caspase-9	GCCTGGTACATCGAGACCTG	GTCCAGCTTCTTGTCTCGGC
GAPDH	AGCCACATCGCTCAGACAC	GCCCAATACGACCAAATCC

Relative gene expression was calculated using the 2^-ΔΔCt^ method, with *GAPDH* as the internal control. Both *GAPDH* and *β-actin* (*ACTB*) were tested for stability under different treatment conditions in Daudi cells. β-actin showed variable expression, especially after curcumin and vitamin D_3_ treatment, while *GAPDH *remained stable. Therefore, *GAPDH* was selected as the reference gene, consistent with previous studies showing its stability in haematological malignancies [14].

Statistical analysis

All experiments were performed in triplicate, and data are presented as mean ± standard deviation (SD). Statistical analysis was conducted using GraphPad Prism (version 8; Dotmatics, Boston, Massachusetts, United States). One-way ANOVA followed by post-hoc analysis was used to compare the various doses of curcumin, vitamin D_3_, and their combination in assessing cytotoxicity and gene expression. A p-value less than 0.05 was considered statistically significant.

## Results

Cytotoxicity with curcumin and vitamin D_3_ in Daudi cells

**Figure 1:**
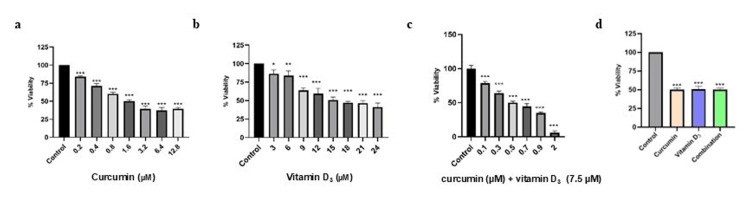
Effect of curcumin (a), vitamin D3 (b), and their combination (c) on Daudi cell viability as measured by MTT assay after 24 hours, and overall percentage viability of Daudi cells following treatment with curcumin (1.6 µM), vitamin D3 (15 µM), and their combination (curcumin (0.5 µM) + vitamin D3 (7.5 µM)) for 24 hours, as compared to untreated control (d) Data are presented as mean ± SD (n = 3). Statistical significance (one-way ANOVA) is indicated: *p < 0.05, **p < 0.01, ***p<0.001 vs. control. MTT: 3-(4,5-dimethylthiazol-2-yl)-2,5-diphenyltetrazolium bromide

Cell viability was assessed by MTT assay (Figure 1) following 24-hour treatment with a range of concentrations of curcumin or vitamin D₃. A 24-hour window period was selected based on the previous studies demonstrating that this interval is sufficient to elicit early apoptotic and cytotoxic effects of curcumin and vitamin D_3_ [15-17]. The results demonstrated a significant, non-linear, dose-dependent increase in cytotoxicity for both drugs (p < 0.001).

The IC₅₀ and IC₂₅ were defined as the concentrations that reduced cell viability by 50% and 25%, respectively, relative to untreated controls [18]. The IC₂₅ concentrations were subsequently used in combination treatments to evaluate potential synergistic effects without inducing excessive cytotoxicity.

With curcumin, the cell viability was found to be 83.25% (0.2 µM), 70.29% (0.4 µM), 60.16% (0.8 µM), 50.15% (1.6 µM), 39.47% (3.2 µM), and 37.94% (6.4 µM,) respectively. Based on this trend, the IC₅₀ value for curcumin was determined to be 1.6 µM±0.16. Likewise, vitamin D_3_ also showed a non-linear dose-dependent cytotoxic effect. Viability declined from 86.4% at 3 µM to 63.88% at 9 µM, 49.47% at 15 µM, 48.05% at 18 µM, and 41.15% at 24 µM. A more substantial reduction was observed at 15 µM±1.12, which was considered the IC₅₀ value for vitamin D₃. Further, for the combination, the IC₅₀ was achieved with an IC₂₅ dose of vitamin D_3_: 7.5 µM and 0.5 µM of curcumin determined from the cytotoxicity assay.

The synergism was quantitatively assessed using the Chou-Talalay method, which calculates the combination index (CI) to evaluate drug interactions (Table 2). A CI value < 1, 1, >1 indicates synergism, additive effect, antagonism, and CI ≈ ∞ or undefined indicates no measurable biological effect. In our study, the CI was calculated as 0.81, indicating a clear synergistic interaction between curcumin and vitamin D₃ at the tested concentrations. This synergy suggests that the two agents may enhance each other’s pro-apoptotic and antiproliferative effects, potentially allowing lower doses of each compound to achieve effective cytotoxicity, thereby reducing potential toxicity while improving therapeutic efficacy [19].

**Table 2:** Combination index of curcumin and vitamin D₃ Combination index of curcumin and vitamin D_3_ at different curcumin combinations (vitaminD_3_ fixed at (IC_25_ dose)7.5 µM) in Daudi cells. CI>1: indicates antagonism, <1: indicates synergism, =1: indicates additive effect. The IC_25_ combination (curcumin 0.5, CI:0.81) is highlighted.

Curcumin (µM)	Vitamin D₃ (µM)	Combination Index (CI)
2.0	7.5	1.75
0.9	7.5	1.06
0.7	7.5	0.93
0.5	7.5	0.81
0.3	7.5	0.68
0.1	7.5	0.56

Nuclear staining with curcumin and vitamin D_3_ in Daudi cells

**Figure 2:**
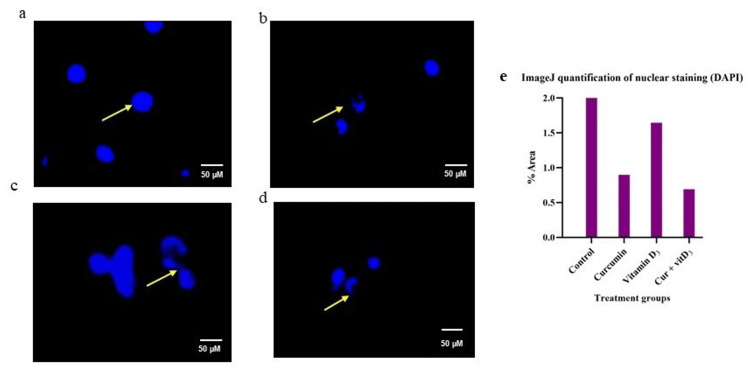
Fluorescence microscopy images of DAPI-stained Daudi cells with curcumin, vitamin D₃, and combination Fluorescence microscopy images of DAPI-stained Daudi cells with (arrows manifest fragmented nuclei with chromatin condensation) untreated control cells (a) appear bright blue with intact nuclei, curcumin (b) -1.6 µM, vitamin D_3_ (c) -15 µM, combination (d) - curcumin-0.5 µM+ vitamin D_3_-7.5 µM displayed condensed nuclei, characteristic of apoptosis and (e) quantification of nuclear stained (DAPI)images, depicted the area(%) of nucleus stained across treatment groups . SAPI: 4′,6-diamidino-2-phenylindole The image is representative of two independent experiments (n = 2). Scale bar = 50 µM

DAPI staining was used to qualitatively visualise nuclear morphology and apoptotic features in treated cells as compared to untreated controls. Images were qualitatively and quantitatively assessed, and representative fields are shown (Figure 2).

Expression of apoptotic and cell cycle regulatory genes

**Figure 3:**
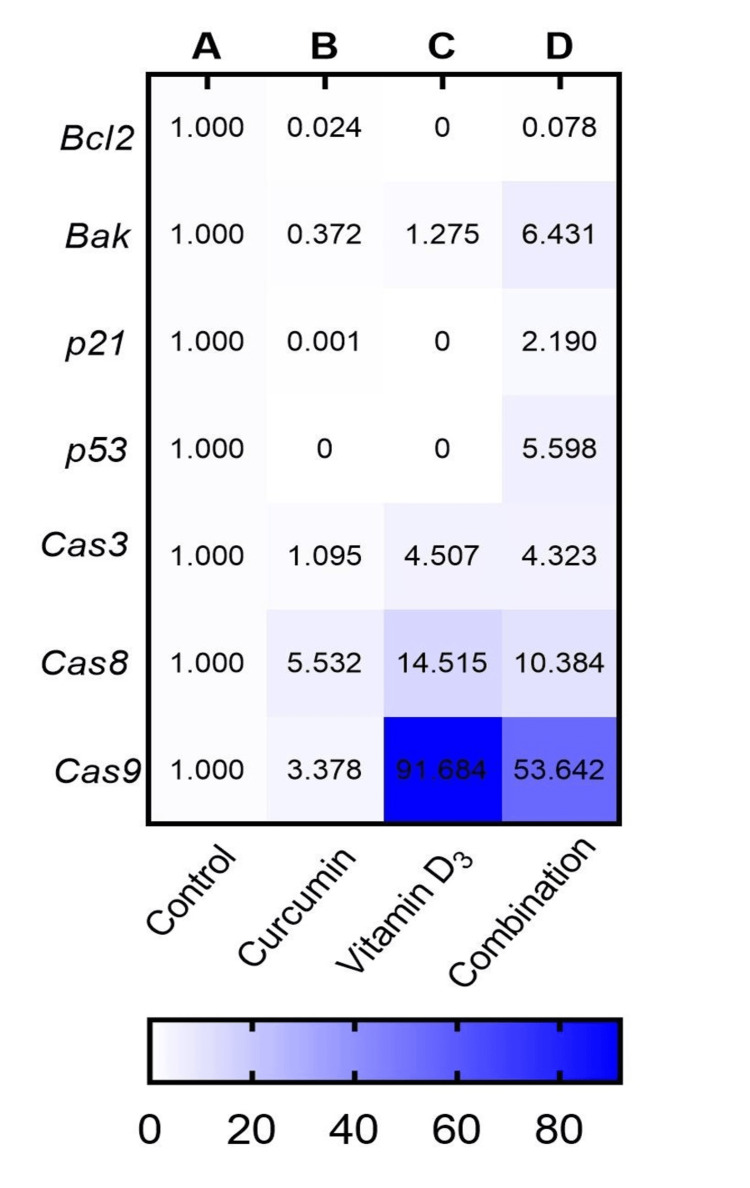
Heatmap showing changes in relative gene expression in Daudi cells after treatment with curcumin, vitamin D₃, and their combination, compared to untreated control Expression levels of apoptosis-related genes (*Bcl-2, Bak, p21, p53, Caspase-3, Caspase-8, Caspase-9*) were measured by RT-PCR and normalized to *GAPDH* using the 2^(-▲▲ct)^ method. The colour intensity represents the fold change in expression relative to control {A: control, B: curcumin (1.6 µM), C: vitamin D₃ (15 µM), D: combination (curcumin-0.5 µM+ vitamin D_3_-7.5 µM)}, with darker blue indicating higher fold change. Data are representative of two independent experiments (n = 2)

**Figure 4:**
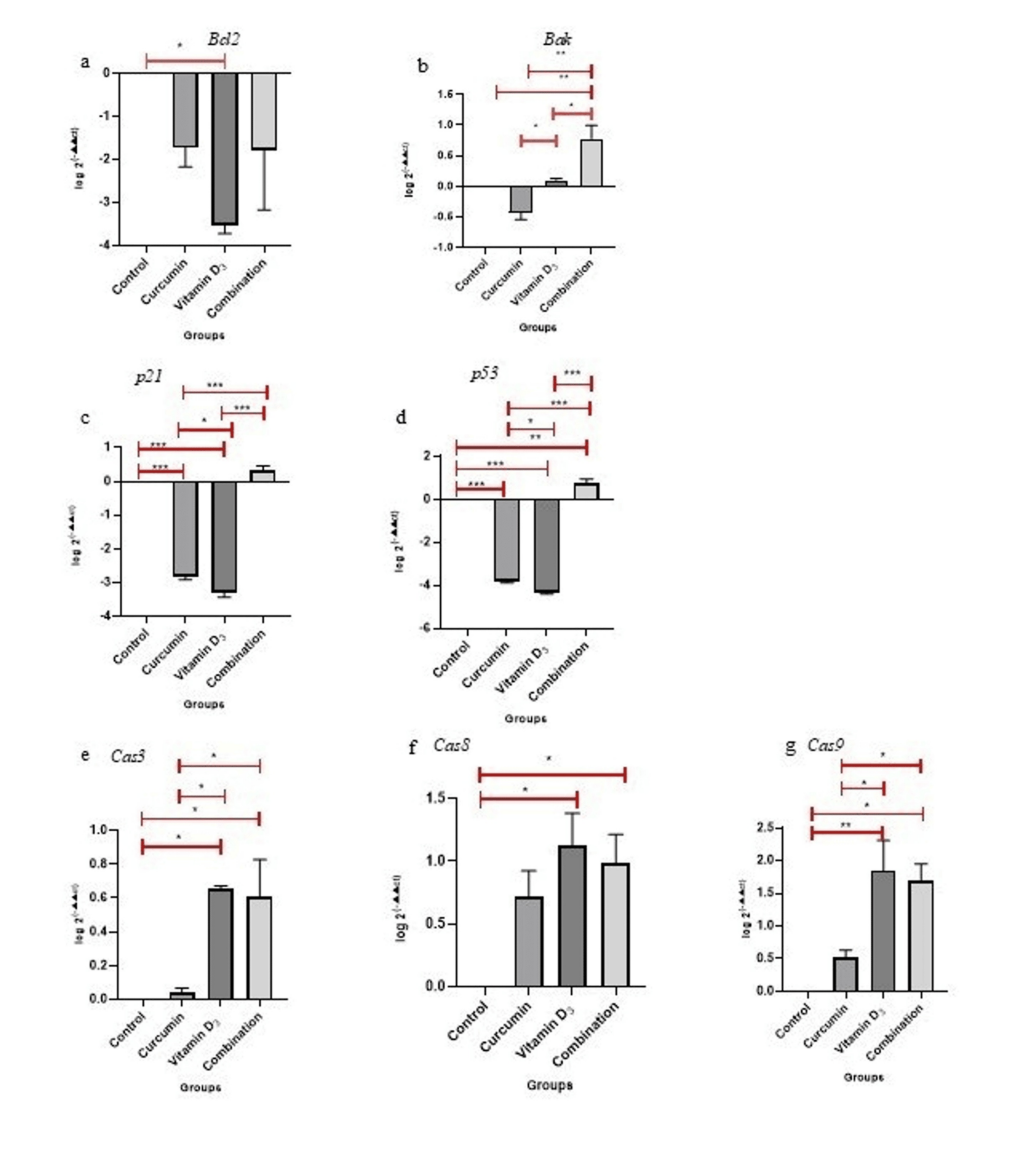
Relative gene expression log [2^(-▲▲ct)] of apoptosis- and cell cycle-regulatory genes in daudi cells treated with curcumin, vitamin D₃ and their combination Relative gene expression log [2^(-▲▲ct)^] of apoptosis- and cell cycle-related genes a. *Bcl2*, b. *Bak* c. *p21*, d. *p53* e. *Cas3*, f. *Cas8* and g. *Cas9* in Daudi cells treated with curcumin (1.6 µM), c- vitamin D₃ (15 µM), and their d-combination (curcumin-0.5 µM; vitamin D₃ -7.5 µM). Data are presented as mean ± SD (n = 2). Statistical significance (one-way ANOVA) is indicated: *p < 0.05, **p < 0.01, ***p<0.001 vs. control.

The effects of curcumin, vitamin D_3_, and their combination on the expression of apoptosis-related genes (*Bcl2, Bak, p21, p53, caspase-3, caspase-8, and caspase-9*) were assessed in Daudi cells using the 2^(-ΔΔCt)^ method. The mean expression values were normalized to the control group (set as 1.0) as presented in Table 3 and Figures 3, 4. The gene expression analysis revealed significant modulation of pro-apoptotic and cell cycle regulatory genes in Daudi cells following treatment with curcumin, vitamin D_3_, and their combination.

**Table 3:** Mean Relative gene expression [2^(-▲▲Ct)] of apoptosis- and cell cycle-related genes in Daudi cells treated with curcumin (1.6 µM), vitamin D3 (15 µM), and their combination (curcumin-0.5 µM+ vitamin D3 -7.5 µM) Expression was normalised to *GAPDH*.

Target genes	Control	Curcumin alone	Vitamin D_3_ alone	Combination
	Mean 2^(-▲▲ct)^			
Bcl2	1.0	0.02	0.0	0.1
Bak	1.0	0.4	1.3	6.4
p21	1.0	0.0	0.0	2.2
p53	1.0	0.0	0.0	5.6
Cas3	1.0	1.1	4.5	4.3
Cas8	1.0	5.5	14.5	10.4
Cas9	1.0	3.4	91.7	53.6

The anti-apoptotic gene *Bcl-2* was significantly downregulated, showing complete suppression in cells treated with vitamin D₃ alone, whereas treatment with curcumin alone or in combination resulted in a non-significant decrease of approximately 0.1-fold. In contrast, the pro-apoptotic gene *Bak* was significantly upregulated with combination treatment (6.4-fold) and showed further increases with curcumin (0.4-fold) and vitamin D₃ (1.3-fold) compared to control, indicating enhanced apoptotic signalling. Cell cycle regulators *p21* and *p53* were significantly upregulated in the combination group as compared to control (2.2-fold and 5.6-fold, respectively), implying synergistic effects on cell cycle arrest mechanisms. Among the caspases, *caspase-3* was significantly upregulated (p<0.05) following treatment with vitamin D₃ (4.5-fold) and with the combination (4.3-fold), with a modest increase observed with curcumin alone (1.1-fold). *Caspase-8*, a key mediator of the extrinsic apoptotic pathway, was significantly elevated (p<0.05) by vitamin D₃ alone (14.5-fold) and the combination treatment (10.4-fold), indicating activation of death receptor-mediated apoptosis. Similarly, caspase-9, which mediates intrinsic (mitochondrial) apoptosis, was significantly upregulated (p<0.05) by vitamin D₃ (2.0-fold), the combination (3.3-fold), and non-significantly increased with curcumin alone (1.5-fold), reflecting engagement of the mitochondrial apoptotic pathway. Collectively, these results suggest that the combination of curcumin and vitamin D₃ enhances apoptotic gene expression more effectively than either compound alone, likely through coordinated activation of both intrinsic and extrinsic apoptotic mechanisms.

## Discussion

This study aimed to explore the cytotoxic and pro-apoptotic effects of curcumin and vitamin D_3_, both individually and in combination, on the Daudi cell line. The Daudi cell line was selected as an in vitro model because it is a well-characterized human Burkitt’s lymphoma cell line that shares key molecular features with aggressive B-cell malignancies, allowing for detailed mechanistic studies of apoptosis. The experimental data from the present study support that the combination of curcumin and vitamin D_3_ has a synergistic effect on cytotoxicity and pro-apoptosis in Daudi cell line by altering the expression of key genes involved in apoptosis and cell cycle regulation.

The MTT assay as shown in Figure 1, revealed a significant, dose-dependent increase in the cytotoxicity of Daudi cells after treatment with curcumin and vitamin D_3_. These results are consistent with previous studies reporting that curcumin inhibits cancer cell growth by affecting several molecular targets, including nuclear factor-kappa B (NF-κB), Akt, and mitogen-activated protein kinases (MAPKs), leading to cell cycle arrest and apoptosis [20]. On the other hand, vitamin D_3_, primarily in its active form 1,25-dihydroxyvitamin D_3_, exerts antiproliferative activity through the vitamin D receptor (VDR), influencing gene expression involved in cell differentiation, immune modulation, and apoptosis [21]. Interestingly, the combination treatment displayed greater efficacy in increasing cytotoxicity than individual treatments, suggesting a potential synergistic interaction. Previous research has demonstrated that curcumin can sensitize cancer cells to other therapeutic compounds, including vitamin D_3_, by modulating oxidative stress, mitochondrial dysfunction, and cell cycle checkpoints [22].

Following the initial assessment of cell viability, DAPI staining was employed to examine nuclear morphology, providing both qualitative and quantitative insights into apoptosis. This technique enabled visualization of characteristic apoptotic features, including chromatin condensation, nuclear shrinkage, and fragmentation. By comparing treated cells with untreated controls, we were able to assess the extent and nature of nuclear changes induced by curcumin, vitamin D₃, and their combination, as illustrated in Figure 2. The staining thus complements functional viability assays by confirming apoptosis at the nuclear level.

Furthermore, RT-PCR was used to assess the expression of pro-apoptotic and anti-apoptotic genes involved in apoptosis (Figures 3, 4). Although *Bcl-2*, an anti-apoptotic gene, was downregulated in all treatment groups, the reduction was statistically significant (p < 0.05) only in the vitamin D₃-treated group. *Bcl-2* overexpression is commonly associated with resistance to apoptosis and poor prognosis in lymphomas [23]. The reduction in *Bcl-2* expression is a key finding, indicating that curcumin and vitamin D_3_ are capable of activating apoptotic pathways in Daudi cells.

Conversely, pro-apoptotic genes such as *Bak* (p<0.001), *caspase-3* (p<0.05), *caspase-8* (p<0.05), and *caspase-9* (p<0.05) were significantly upregulated, particularly in the combination group (curcumin and vitamin D_3_). These genes are central to the execution of apoptosis. *Caspase-9* is involved in the intrinsic (mitochondrial) pathway, while caspase-8 mediates the extrinsic (death receptor-mediated) pathway. Both converge on *caspase-3*, the executioner caspase responsible for the cleavage of numerous cellular substrates leading to apoptotic cell death [24]. The upregulation of these caspases supports the activation of both apoptotic pathways in our combination treatment.

Following the apoptosis genes, *p53*, a tumour suppressor often referred to as the "guardian of the genome," plays a critical role in initiating apoptosis in response to cellular stress, DNA damage, or oncogene activation [25]. Its downstream effector, *p21*, inhibits cyclin-dependent kinases (CDKs), resulting in G1-phase cell cycle arrest and preventing proliferation of damaged cells [26]. The simultaneous upregulation of both genes highlights the reactivation of cell cycle checkpoints and supports the pro-apoptotic activity observed in curcumin- and vitamin D_3_-treated cells.

In the present study, as shown in Figure 3, treatment of Daudi cells with either curcumin or vitamin D₃ alone did not increase *p53* or *p21* expression. This may be attributed to the intrinsic characteristics of Daudi cells, which are Epstein-Barr virus (EBV)-positive and exhibit attenuated *p53/p21* signalling due to viral interference with *p53* transcriptional activity rather than gene mutation [27,28]. EBV-encoded proteins such as EBNA-3C and LMP-1 have been reported to suppress *p53* function and downstream *p21* activation [29].

But, when curcumin and vitamin D₃ were applied in combination, both *p53* and *p21* were markedly up-regulated, suggesting that this dual treatment may overcome the basal repression of the *p53* pathway in Daudi cells. Curcumin has been shown to activate *p53* and its downstream effector *p21* through oxidative stress and mitochondrial perturbation [30], while vitamin D₃ can modulate *p53* signalling through vitamin D receptor (VDR)-mediated cross-talk [31]. The combination might therefore synergistically relieve EBV-mediated repression by promoting both transcriptional activation (via VDR-p53 interaction) and post-translational stabilisation of *p53* (via curcumin-induced stress response), ultimately restoring downstream *p21* expression and cell-cycle arrest. These findings support the hypothesis that combinatorial approaches can re-engage silenced tumour-suppressor pathways in lymphoma cells, offering potential therapeutic relevance in contexts where *p53* signalling is functionally suppressed but not genetically deleted.

Based on the findings from the present study, vitamin D₃ activated the extrinsic apoptotic pathway, as evidenced by a 14.5-fold increase in *caspase-8* expression, the highest among all treatment groups for this gene. *Caspase-8* is a well-characterized initiator of extrinsic (death receptor-mediated) apoptosis, and its upregulation here strongly supports death receptor pathway engagement. While *caspase-9* was also elevated (91.7-fold), this increase likely reflects crosstalk from extrinsic signalling amplifying the intrinsic pathway via Bid cleavage, a mechanism previously described in hematologic malignancies treated with vitamin D analogs [32]. The moderate induction of *Bak* (1.28-fold) further supports secondary engagement of the intrinsic mitochondrial pathway. This dual-pathway activation by vitamin D₃ aligns with previous findings, where vitamin D compounds were shown to activate the intrinsic pathway [33,34] as well as Fas/FasL signalling by modulating *Bcl-2* family proteins, leading to both death receptor and mitochondrial apoptosis [35].

In contrast, curcumin primarily induced the intrinsic (mitochondrial) apoptotic pathway. This is supported by the increased expression of *caspase-9* (3.4-fold) and elevation of *Bak* (0.37-fold), both key components of mitochondrial apoptosis. The increase in *caspase-3* (1.1-fold), an executioner caspase activated downstream of mitochondrial signals, further reinforces this conclusion. Notably, *caspase-8* expression was relatively unchanged (5.5-fold vs. 14.5-fold in vitamin D₃), suggesting limited involvement of extrinsic signalling. These results are consistent with the established role of curcumin in disrupting mitochondrial membrane potential, promoting cytochrome c release, and activating *caspase-9*-mediated apoptosis in various cancer types, including leukaemia [36-38].

Further, the combination of curcumin and vitamin D₃ led to potent activation of both intrinsic and extrinsic apoptotic pathways, as well as cell cycle arrest mechanisms. This group exhibited high levels of *caspase-8* (10.4-fold), *caspase-9 *(53.6-fold), *caspase-3* (4.3-fold), and *Bak* (6.4-fold), indicating coordinated activation of upstream and downstream apoptotic machinery. In the present study, treatment of Daudi cells with either curcumin or vitamin D₃ alone did not result in appreciable induction of *p53* or *p21* expression.

Taken together, these findings suggest that curcumin and vitamin D₃, particularly in combination, effectively engage and amplify both intrinsic and extrinsic apoptotic mechanisms, while also promoting cell cycle arrest through *p53* and *p21.* Mechanistically, curcumin has been reported to enhance vitamin D receptor (VDR) expression and transcriptional activity, thereby potentiating vitamin D₃-mediated gene regulation [39,40]. This interaction may sensitise cells to vitamin D₃ by strengthening VDR-dependent apoptotic and cell cycle pathways. Such convergence on shared molecular targets provides a plausible basis for the observed synergistic pro-apoptotic and cytotoxic effects. Further studies involving in vivo validation and dose optimisation will be necessary to determine clinical relevance and therapeutic windows.

Despite the potential findings, this study has some limitations. Firstly, it is an in vitro study limited to mRNA-level analysis of apoptosis-related genes. Protein-level validation (e.g., via Western blot or immunocytochemistry) was not performed, which could have provided additional confirmation of the observed gene expression changes, and also the results observed in cell lines may not always translate directly to in vivo systems due to the complexities of tumour microenvironments and systemic metabolism. Secondly, the study was conducted using only a single Daudi cell line and did not include a normal (non-cancerous) cell line or a positive control. This restricts the assessment of treatment specificity, comparative efficacy, and generalizability to other lymphoma subtypes or hematologic malignancies. Nevertheless, the primary aim of this study was to provide mechanistic insights into the cytotoxic and pro-apoptotic effects of curcumin and vitamin D₃ in B-cell lymphoma cells. Future studies are planned to include additional cell lines and appropriate controls to validate and extend these findings, thereby strengthening the translational relevance of the results. Additionally, we did not evaluate the pharmacokinetics, bioavailability, or potential toxicity of curcumin and vitamin D_3_, which are crucial parameters for clinical application. Finally, gene expression was assessed at a single dose level, and detailed dose-response relationships were not established. Future in vivo experiments and clinical trials are necessary to confirm the therapeutic potential and safety of these compounds in combination.

## Conclusions

The present study demonstrates that both curcumin and vitamin D₃ exert cytotoxic and pro-apoptotic effects on human non-Hodgkin lymphoma Daudi cell lines. Their combination showed synergistic effects on cytotoxicity, pro-apoptosis, and expression of genes involved in apoptotic signalling and cell cycle regulation, such as *Bcl-2, Bak, caspase-3, caspase-8,* *caspase-9, p53*, and *p21*. This supports the concept that using natural compounds in combination may enhance therapeutic efficacy while minimising toxicity compared to conventional chemotherapeutics. Yet, further studies are warranted in animal models and additional B-cell lymphoma lines to evaluate the in vivo relevance and pharmacokinetics of this combination strategy.
